# Managing uncertainty in advanced liver disease: a qualitative, multiperspective, serial interview study

**DOI:** 10.1136/bmjopen-2015-009241

**Published:** 2015-11-19

**Authors:** Barbara Kimbell, Kirsty Boyd, Marilyn Kendall, John Iredale, Scott A Murray

**Affiliations:** 1 Primary Palliative Care Research Group, Centre of Population Health Sciences, The University of Edinburgh, Edinburgh, UK; 2 Royal Infirmary of Edinburgh, Edinburgh, UK; 3 The University of Edinburgh, Edinburgh, UK

**Keywords:** PALLIATIVE CARE, QUALITATIVE RESEARCH

## Abstract

**Objective:**

To understand the experiences and support needs of people with advanced liver disease and those of their lay and professional carers to inform improvements in the supportive and palliative care of this rapidly growing but currently neglected patient group.

**Design:**

Multiperspective, serial interviews. We conducted up to three qualitative in-depth interviews with each patient and lay carer over 12 months and single interviews with case-linked healthcare professionals. Data were analysed using grounded theory techniques.

**Participants:**

Patients with advanced liver disease of diverse aetiologies recruited from an inpatient hepatology ward, and their lay carers and case-linked healthcare professionals nominated by the patients.

**Setting:**

Primary and secondary care in South-East Scotland.

**Results:**

37 participants (15 patients, 11 lay and 11 professional carers) completed 51 individual and 13 joint patient-carer interviews. Nine patients died during the study. Uncertainty dominated experiences throughout the course of the illness, across patients’ considerable physical, psychological, social and existential needs and affected patients, lay carers and professionals. This related to the nature of the condition, the unpredictability of physical deterioration and prognosis, poor communication and information-sharing, and complexities of care. The pervasive uncertainty also shaped patients’ and lay carers’ strategies for coping and impeded care planning. While patients’ acute medical care was usually well coordinated, their ongoing care lacked structure and focus.

**Conclusions:**

Living, dying and caring in advanced liver disease is dominated by pervasive, enduring and universally shared uncertainty. In the face of high levels of multidimensional patient distress, professionals must acknowledge this uncertainty in constructive ways that value its contribution to the person's coping approach. Pervasive uncertainty makes anticipatory care planning in advanced liver disease challenging, but planning ‘just in case’ is vital to ensure that patients receive timely and appropriate supportive and palliative care alongside effective management of this unpredictable illness.

**Strengths and limitations of this study**

- This is the first in-depth serial interview study to explore people's experiences of advanced liver disease.
- The multiperspective approach facilitated comparing and contrasting the experiences of patients, lay carers and professionals, while serial interviewing was key to illuminating the complex and evolving needs and experiences of patients and families.
- As patients were recruited in a single locality and from a specialist inpatient liver unit, their care experiences may differ from other areas and those of patients accessing other hospitals.
- Given the current absence of a single agreed definition of advanced or end-stage liver disease, we developed inclusion criteria through an expert consensus of when a palliative care approach should be appropriate in advanced liver disease.
- The findings have implications for identifying and planning optimal supportive and palliative care in advanced liver disease, which could be tested in a clinical trial.

## Introduction

Chronic liver disease is the third commonest cause of premature death in the UK and a rapidly growing problem.^1^ Its leading causes are alcohol misuse, metabolic syndromes linked to obesity and hepatitis B and C. Chronic liver disease affects younger people compared with heart, lung or kidney failure. In England, more than 1 in 10 of deaths of individuals in their 40s are linked to liver disease.^2^

Comorbidities, the underlying causes of liver disease and limited donor organ supplies make liver transplantation unavailable for a large number of patients. Many thus stand to benefit from early access to appropriate supportive and palliative care.^3^ While access to specialist palliative care services for people with non-malignant disease has doubled in the past decade,^4^ inequalities are still stark.^5^

Evidence-based guidance for the supportive and palliative care of people with advanced liver disease, similar to that already available for other non-malignant end-stage disease, is urgently needed. People with advanced liver disease face many physical and psychosocial challenges, but in-depth research which considers their unique care and support needs is lacking.^6^ We aimed to understand the experiences, needs and priorities of patients and their lay and professional carers towards the end of life to propose effective models of care in advanced liver disease.

## Methods

A constructivist theoretical perspective and qualitative, multiperspective, serial in-depth interviews were chosen to meet the aims of the study best.^7^ ^8^ Constructivism takes a relativist perspective, which considers the illness experience an inherently subjective experience as represented by individual, context-bound accounts. Rather than seek to verify scientific ‘truth’, it focuses on discovery and the creation of a deeper understanding of phenomena through naturalistic methodologies and the viewpoint of those whose experiences are the object of investigation.^9^ A constructivist approach therefore responded well to the broad, exploratory intentions of this research.

A patient and carer advisory group provided support and guidance throughout the project.

### Recruitment

Supported by clinical staff, we recruited patients presenting with ascites from an inpatient liver unit between October 2011 and June 2012. Ascites is the most common complication of advanced liver disease and an indicator of decompensation.^10^ Ascites also indicates a poor prognosis, with an expected mortality of around 50% at 2 years.^11^ We excluded patients who had non-hepatic cancer or other long-term conditions as their primary diagnosis, patients listed for liver transplantation and those with significant cognitive impairment. Sampling was purposive to achieve a spread across aetiologies and sociodemographics. Thirty-eight patients were invited to participate, of whom 25 agreed. Ten were withdrawn before their first interview due to declining health, death or loss of contact. Nine patients died during the study. We asked patients to nominate the lay and professional carers most involved in their care. Eleven lay carers took part in the study. Eleven professionals, nominated by 10 patients, were also interviewed.

Written consent was obtained before each interview, and participants reminded that they could pause or terminate the interview at any time. Ongoing verbal consent was also ascertained at every patient contact (face-to-face or telephone). Lay and professional carers were asked separately for their consent.

### Data generation

Demographic information was obtained from each participant's medical records. We calculated the Model of End-Stage Liver Disease (MELD) score as a measure of liver disease severity and a deprivation category (DEPCAT) score for socioeconomic status.

We interviewed patients and lay carers up to three times over 12 months. Interviews were conducted individually or jointly according to participants’ wishes. Patients were telephoned bi-monthly by the researcher so that follow-up interviews could be arranged flexibly to capture key changes in the person's condition or circumstances while being sensitive to the needs of the participant at that time. Interviews were conducted by an experienced qualitative researcher (BK) and lasted 30–150 min. Most patient and carer interviews took place in the patient's or carer's home, a small number were conducted in a quiet room on the hospital ward. One-off interviews with case-linked professionals were conducted to contextualise the patient and carer data. Professional interviews were conducted face-to-face or by telephone as preferred to further encourage participation. All interviews were audio recorded and detailed field notes kept to contextualise the interview data.

Patients and lay carers were asked about their experiences of liver disease, their current physical, psychosocial, existential and information needs and their thoughts about the future. Follow-up interviews explored evolving needs and the adequacy of services and support provided. Bereavement interviews with lay carers explored the patient's terminal phase of illness and death, and the support and care offered then. Interviews with professionals sought their views, experiences and perceived challenges in providing effective care. Interview guides were used flexibly to allow participants to express their greatest concerns.

### Data analysis

All interviews were transcribed and analysed by BK aided by qualitative data management software QSR NVivo V.9. Data analysis drew on constructivist grounded theory and its techniques of coding, constant comparison and memo-writing.^12^ It involved an iterative process of separating, sorting and synthesising the data, leading from generic substantive coding to increasingly abstract and theoretical coding. Codes and categories were compared to identify differences and similarities, and refine and modify codes. Contextual information from the field notes as well as regular discussions with the multidisciplinary research team and the study's lay advisory group added depth and breadth to data interpretation.

The data were analysed: (1) by participant group (patients, lay carers or professionals), (2) by ‘integrated case’ (case-linked patient/carer/professional triads) and (3) longitudinally to illuminate participants’ evolving needs. This analysis was informed by a menu of questions designed to stimulate the exploration of change.^13^ Comparisons across these data sets allowed consideration of commonalities and differences between groups, cases and over time.

### Quality assurance

A detailed audit trail was recorded to facilitate quality assessment of the study conduct.^14^ Procedural decisions taken during the recruitment and data generation phases of the research were recorded in extensive field notes. An analytical decision trail was documented in NVivo through a detailed data analysis journal, copies of the coding structure at different time points and time-stamped memos showing evolving analytical ideas about cases, codes and categories. The study conduct met COREQ criteria for reporting qualitative studies.^15^

## Results

We recruited 15 patients, 11 lay carers and 11 case-linked professionals. There were seven male and eight female patients with an average age of 58.8 (range 35–84). Their primary disease aetiologies were: alcohol-related liver disease (ALD), non-alcoholic fatty liver disease (NAFLD), hepatitis C (HCV), primary hepatocellular carcinoma, autoimmune hepatitis and cryptogenic liver disease. The mean MELD score at recruitment was 16.3 (range 9–26). Nine patients lived with their lay carers, six lived alone. The case-linked professionals comprised eight general practitioners and one consultant hepatologist, hospital-based alcohol liaison nurse and community palliative care nurse each. Fifty-one individual interviews were conducted, 13 of these jointly with patient and lay carer. Three carers agreed to bereavement interviews. Table 1 gives an overview of the participants and interviews.

**Table 1:** Overview of patient participants and interviews conducted

Patient	Aetiology	DEPCAT	MELD at recruitment	Months in study	Admissions during study involvement	Interviews per time point (P=patient; C=carer; P&C=joint)	Status at end of study
Male, 68	ALD	4	14	12	0	P&C1, P&C2, P&C3	Alive
Female, 38	ALD, HCV	5	20	12	3	P1, P2, P3, C1, C2, C3; GP	Alive
Female, 66	AIH	4	20	5	5	P1, P2	Dead
Female, 60	ALD	5	17	7	6	P1, P2, C1, Bereavement; Consultant	Dead
Female, 84	NAFLD	4	9	11	13	P1, P2, P3	Dead
Male, 74	HCC *(NAFLD)*	7	17	1	0	P1, Bereavement; GP	Dead
Male, 77	ALD *(NAFLD)*	6	9	9	21	P1, P&C2, P&C3; GP; Palliative Care Nurse	Dead
Male, 75	NAFLD	3	21	6	9	P1; GP	Dead
Male, 35	ALD	4	24	12	5	P1, P2, P3, C1, C2; Alcohol Liaison Nurse	Alive
Male, 58	ALD	5	13	6	7	P1	Dead
Female, 41	Cryptogenic	3	10	8	5	P&C1, P2, C2, P&C3; GP	Alive
Female, 56	ALD, HCV	4	13	6	0	P1	Alive
Female, 45	ALD	3	16	7	2	P1, P2; GP	Dead
Female, 68	NAFLD	3	15	5	9	P&C1, Bereavement; GP	Dead
Female, 37	ALD	7	26	9	2	P&C1, P&C2, P&C3; GP	Alive

(DEPCAT (Deprivation Category index): 1=least deprived; 7=most deprived).

AIH, autoimmune hepatitis; ALD, alcohol-related liver disease; GP, general practitioner; HCC, hepato-cellular carcinoma; HCV, hepatitis C virus; MELD, Model of End-Stage Liver Disease; NAFLD, non-alcoholic fatty liver disease.

### Overview of findings

The extensive physical and psychological burden in advanced liver disease described previously was evident.^6^ The key factor which emerged as unifying the experiences of all participants however was pervasive uncertainty. Uncertainty is described as a person's inability to extract meaning from illness-related events.^16^ This occurs “when details of situations are ambiguous, complex, unpredictable or probabilistic; when information is unavailable or inconsistent; and when people feel insecure in their own state of knowledge or the state of knowledge in general” (ref. 17, p. 478). We describe how uncertainty dominated all participants’ accounts and defined their experiences.

### Ambiguous onset and confused understanding of the disease

Patients’ experiences of the onset of their illness were characterised by the insidious nature of their early symptoms and uncertainty about whether to consult their general practitioner (GP), often resulting in the diagnosis of liver disease at an advanced stage.

I didn't feel particularly well, but I couldn't tell you why I didn't feel well. I just had put it down to the fact I was a bit stressed out. (Patient 11, female, 41, first interview)

Receiving a diagnosis was a similarly ambiguous event, magnified by difficulty understanding the professional language. Use of the word ‘cirrhosis’ was particularly confusing for those with aetiologies other than alcohol excess, as people tended to associate the term with this cause.

Patient: They just told me that I had liver…they called it another name, it sort of fooled me for a wee while. (…)

Interviewer: Cirrhosis? (…)

P: That was it. And it wasn't until I seen it printed in a letter they sent me, when I got the letter that's when I realised it was [primary liver] cancer.

(Patient 6, male, 74, first interview)

Patients’ physical experiences described a gradual decline punctuated by unpredictable but relatively short-lived episodes of decompensation (eg, ascites) and more dramatic acute events (eg, gastrointestinal bleeding), which both required inpatient treatment. Patients felt satisfied with the acute medical care they received on such occasions. The problems that most often disrupted daily life were fatigue and ascites. Many patients also reported intermittent problems with mobility and balance. A lack of understanding of the cause of these problems and their unpredictable occurrence fostered more uncertainty. Several people recounted incidents of falling, making them lose confidence in their abilities and restrict their activities to avoid further falls. Extensive, complex and ever-changing medication regimens were a further source of uncertainty for patients and caregivers alike.

Regardless of aetiology, most patients had had little knowledge of liver disease prior to their diagnosis, reflecting a general lack of public discourse about this condition. This was reinforced by a perceived lack of education as part of their ongoing medical care. As a result, patients were unsure which physical symptoms to ascribe to their liver disease.

This lack of energy thing, I mean, is that quite normal for somebody who's got liver disease (…) or am I just milking it a bit? Should I be ok or what? I don't know what all this is about. (Patient 11, female, 41, first interview)

Clarity about their situation was further hampered by conflicting information received from the different professionals involved in their care. At the same time, patients’ poor understanding of their condition left them unsure what to ask professionals. Participants were particularly poorly informed about what to expect from their illness and how it might progress in the future, leaving them unsure how to cope and plan.

Somebody should be able to sort of say, “Well this will happen sometime or that will happen sometime,” and you know, warn you what it's going to do and what you've got to do when it happens. (Patient 8, male, 75, first interview)

However, not everyone felt that more specific information was required. Preparing patients and their families for the realities of living with an illness as unpredictable and uncertain as advanced liver disease was in itself considered empowering by some.

People get frustrated about not knowing what's going on (…), but if you say right at the onset this is what's going to happen, it's going to be a big rollercoaster, it is going to be full of ups and downs, there's going to be lots of times of nothing happening, people will come and change opinions by the second and that's what is literally going to happen, be ready for it. (…) That in itself for me is understanding what's going on. (Lay carer 11, second interview)

### Uncertainty as a feature of everyday life

Employment was particularly important for this younger group of people. Inability to work caused financial pressures, with some patients becoming increasingly dependent on their families. Their caregivers felt under similar pressure. Those with alcohol-related disease were preoccupied with the challenges of overcoming their dependency, which added to their psychological burden. Participants were acutely aware of the stigmatising reputation of liver disease and its popular association with alcohol misuse. Participants adopted strategies for avoiding potentially stigmatising situations, such as eschewing social contact or discussion of their condition.

Increasing physical complications distressed participants, realising that recovery was unlikely and fearing future deterioration. Despite expressions of acceptance of their situation, low mood was pervasive.

Sometimes I lie in the room and cry and just think about things, you know, things that you could remember that you could do and now you can't. (Patient 4, 60, first interview)

Patients and their lay carers tended to deal with the uncertainty of the situation by simply ‘getting on with it’ and hoping for the best. This indicated a deliberate cognitive and emotional distancing to enable better coping. A key strategy for maintaining a positive mind-set was to put faith in healthcare professionals’ abilities and commitment.

A lack of awareness of what types of support were available to them meant that many patients and lay carers struggled to articulate what support they would value most. Professionals similarly showed a lack of awareness of support services.

I don't know of many support services. That's a bit of a worry actually, considering that I'm the last point of contact for people. (…) Are there any specific services for folks with liver conditions? I don't know any of them if there are any. (Community palliative care nurse)

### Inadequate doctor–patient communication and discontinuity of care

Patients considered the time available during appointments too limited for effective information exchange. They also criticised hospital staff for regularly approaching them and advising on their care without making their role and authority to do so clear enough. This left patients and families unsure about where these instructions were coming from. Several participants also felt that explanations were often too medicalised.

What they're saying is, “Right, your potassium level's at such and such, your sodium level's at such and such,” what else, “your blood count was this, your blood pressure was that.” (…) and they don't actually tell me what it means. It is good? Is it bad? Is it happy medium? I don't know. (Patient 15, female, 37, second interview)

Relational and organisational continuity of care were consistently inadequate in primary and secondary care settings. This added another dimension of uncertainty. Being able to see the same GP who knew about their condition varied across participants and many lacked continuity of care with their designated consultant. Many participants also remarked on the frequent turnover of hospital nursing and clinical staff.

Follow-up care was delivered through outpatient appointments and the monitoring of bloods commonly undertaken in primary care. However, attendance at follow-up appointments was often impeded by illness-related factors, lack of support and repeated hospitalisation. Some participants also questioned the usefulness of these appointments. Gaps between hospital appointments were also considered unhelpfully long.

It's just basic. They just ask how I've been feeling, have I had a drink, are you eating well? And then they'll put you on the bed and have a wee prod about and then take your blood and that's it. (Patient 9, male, 35, second interview)

What tended to happen was [patient] would be in hospital very unwell, have fluid, his ascites drained off and then he'd come out and I'd say, “So when's your next follow-up appointment?” and he would say, “Oh, I'm seeing [consultant] in three or four months.” And to me that didn't seem appropriate. (…) It seemed far more appropriate that he was seen on a more regular basis (GP8).

The experiences of care as described above were not found to change over time.

### Uncertainty as a barrier to talking about and planning for deteriorating health and dying

Giving and receiving a prognosis was beset with uncertainty. GPs felt they lacked expertise and confidence and thought conversations about progression and prognosis should be conducted by specialists. Patients also differed in the extent to which they wished to know their prognosis. As a result, prognosis was rarely discussed.

Most professionals interviewed had not engaged in advance care planning with patients with liver disease, citing difficulty in pinpointing the appropriate time to start this. Additionally, specialists’ tendency to continue interventions until the final stages of the illness left GPs unsure about the status of their patient's condition and thus when to consider them for palliative care.

Interviewer: Was there any particular point or event from where you felt that she needed a palliative care approach or maybe to put her on the [palliative care] register?

GP14: No, not particularly, you know? I mean they kept transfusing her, so they didn't say, “Stop transfusing her.”

Specialists however tended to address this issue with patients only when their terminal phase was evident, by which point conversations could be difficult to conduct.

Interviewer: What would normally be your trigger point for initiating those sort of conversations?

Consultant: Well, I think when you are clear people are dying, you know? (…) The difficulty with these patients is that often by the time that you know they are dying they're often too confused or unwell or sleepy to actually have that conversation so, you know, it can be difficult.

GPs’ reluctance to contradict specialist colleagues and uncertainty regarding their own place in a patient's care constituted further barriers.

Maybe I should have written a letter to the consultant to say, “Look, we're treating this as palliative care, is it possible to step back on the thing?” But somehow you don't feel that it's your place to say that, because they're under the care of a liver specialist who obviously knows what they're doing. (GP7)

Finally, there was consensus among all the professionals that palliative care for people with liver disease deserved more attention. Several GPs mentioned the usefulness of specialist nurses who support people with other conditions such as heart failure, and were unsure whether these existed for those with liver disease.

## Discussion

### Summary

Pervasive and enduring uncertainty throughout the course of the condition defined and unified the experiences of patients, lay carers and professionals. This related to the vague and unpredictable nature of the disease, limited understanding of the condition and its likely progression, lack of effective communication and discontinuity of care. Increasingly frequent, debilitating complications brought acute episodes of physical and psychosocial distress in the context of a gradual overall decline in health. While patients’ acute medical care appeared to be largely well-coordinated, their ongoing care in the community lacked structure and focus. Forward planning was largely absent. Care was symptom-focused, episodic and reactive, in primary and secondary care settings, and was consequently poorly aligned with the long-term, multidimensional needs of this patient group. GPs appeared disempowered due to a lack of experience, confidence and being uncertain about their role in these patients’ care.

### Comparison with existing literature

Patients experienced an erratic trajectory of physical and psychosocial distress similar to that reported in heart failure and chronic obstructive pulmonary disease.^18^ ^19^ This in-depth, serial interview study of people with advanced liver disease is the first to describe the impact of insufficiently coordinated care that was largely focused on disease management in hospital. Patients with advanced heart and respiratory diseases often benefit from the support of specialist nurses and allied health professionals,^20^ ^21^ but such services are not available for those with liver disease in general. In addition to debilitating physical complications and pervasive medical uncertainty, which together created a substantial and enduring psychological burden, patients and lay carers also faced several personal and social sources of uncertainty. This mirrors previous research in this patient group.^22^ ^23^ Participants recounted instances of enacted and perceived stigma in their clinical care and everyday life, which paralleled the patient experience in lung cancer and previous accounts of liver disease.^24–26^

### Implications for managing inherent uncertainty in advanced illness

Uncertainty is characterised by ambiguity, vagueness, unpredictability and incomplete information.^27^ Osler declared that, “Medicine is a science of uncertainty and an art of probability.”^28^ Skill in dealing with uncertainty is therefore an art professionals must learn and share with patients and lay carers. Being explicit about the uncertain nature of the condition may in itself reduce patients’ anxieties.^29^ After all, Hippocrates stated that the ability to forecast the future course of the illness, including the uncertainties, is what is most appreciated by patients.^30^ Being aware of inevitable uncertainty is important and empowering and likely to help people live as well as possible with deteriorating health.

Uncertainty can challenge a person's ability to cope with and adapt to chronic illness.^31^ In this study, possibly due to a lack of scope for reducing uncertainties, patients principally sought to manage uncertainty based on their assessment of whether having certainty about their situation would empower or undermine them.^32^ Living with uncertainty can also a preferred state for some patients with chronic obstructive pulmonary disease.^19^ This confirms that reducing uncertainty is not necessarily a person's desired goal.^33^

Psychosocial support is key to the successful management of uncertainty and thus better coping, but was notably absent. Uncertainty proved a key barrier to advance care planning and consequently hindered the labelling of patients as ‘palliative’. This mirrors, but we found was more pervasive, than in other organ failure conditions and cancer.^34–36^ Continued hospital treatments, even where those were palliative in nature such as blood transfusions and paracentesis, contributed to GPs’ uncertainty as to the appropriateness and timing of mentioning a palliative care approach. The erratic and unpredictable trajectory of advanced liver disease means that the focus should be shifted from asking, “*Is it time for a palliative care approach yet*?” to considering the problems faced by patients and families as a whole as their illness progresses, what their goals and needs are and who can help with those most effectively.^37^ Moreover, participants trusted healthcare professionals to have their best interests at heart. Trust and confidence in one's healthcare provider is not only key to reducing uncertainty in the ill person,^16^ but can offer a sound basis for introducing anticipatory care conversations.

### Implications for practice and research

Patients and their families need better education and information about the typical features of advanced liver disease. Promoting public awareness may lead to earlier diagnosis and less prejudice. Ongoing opportunities to seek information and ask questions are needed throughout the illness. Professionals should be open with patients about the uncertain prognosis and progression. This may also facilitate anticipatory care planning by encouraging the idea of ‘hoping for the best, but preparing for the worst’. Ascertaining patients’ individual perceptions of uncertainty enables information-sharing and interventions that complement that person's approach and thus support coping.

Despite patients voicing concern about their GPs’ lack of expertise in liver disease, there may be little value in developing their specialist knowledge.^38^ Participants considered their medical care to be largely satisfactory, but lacked coordinated care and focused psychosocial support. Primary care teams have expertise in the holistic care and management of people with advanced long-term conditions, but this works best if patients are identified and primary and secondary care services collaborate and communicate effectively so that important conversations and care plans are shared.

Continuity in care relationships was poor in primary and secondary care settings, and inpatient and outpatient care. Access to a single co-ordinating care professional can help avoid unplanned hospitalisation and is key to good end-of-life care.^39^ ^40^ The utility of a community liaison liver nurse specialist should be evaluated. This nurse might take the lead in managing and co-ordinating patients’ ongoing care in the community, which would address several of the identified shortcomings, and liaise with other services, such as specialist palliative care, when needed. Box 1 provides a summary of proposed service improvements.

Box 1 **Practical suggestions for clinicians caring for patients with advanced liver disease and their lay carers**

- Explain how the patient's symptoms may relate to the illness (eg, lack of energy) and explain about other common problems they may encounter.
- Acknowledge the inherent uncertainties in the condition, and involve patient and carer in making a plan in advance to deal with these uncertainties.
- Be mindful of patient-friendly communication: introduce yourself, avoid ambiguous language or medical jargon, provide opportunities to ask questions and clarify issues.
- Build on the patient's current understanding and individual approach to coping with uncertainty.
- Discuss wider life circumstances and assess for and address low mood.
- Build on patient and carer trust in healthcare professionals to encourage anticipatory care conversations; revisit these frequently.
- Identify and promote support services/resources available to liver patients and their carers.
- Strengthen communication with palliative care services and other allied professions to maximise their involvement.
- Strengthen communication with members of the patient's healthcare team across settings to ensure complementary and timely follow-up.
- Promote public understanding of liver disease, raising awareness about its insidious nature and dispelling myths to reduce its stigmatising reputation.

#### Strengths and weaknesses of the study

This is the first in-depth serial interview study to explore people's experiences of advanced liver disease outside of transplantation. A key strength of this study was its methodology. The inclusion of a spectrum of ages, genders and disease aetiologies served to highlight the relative uniformity of the patient experience in advanced liver disease. Similarly, the multiperspective approach facilitated comparing and contrasting the experiences of patients, lay carers and professionals. Serial interviewing was key to illuminating the complex and evolving needs and experiences of these patients and families. Our bi-monthly telephone calls enabled us to ensure the interviews captured the dynamic events and experiences.

Patients were recruited in a single locality in the UK and from a specialist inpatient liver unit. Their care experiences may therefore differ from other areas and those of patients accessing other hospitals. In the absence of a single agreed definition of advanced or end-stage liver disease, the inclusion criteria constituted an empirical measure that may not be representative of this patient population at large. Asking patient participants to nominate the professionals to be interviewed meant an absence of control over the type of professions that would be represented, resulting in a sample dominated by GPs. Ethnic minorities were missing from the study sample.

## Conclusion

Living, dying and caring with advanced liver disease is dominated by pervasive, enduring and universally shared uncertainty. Uncertainty not only defines everyone's experiences, but proves a key mediating factor in their actions. Acknowledging the inevitable uncertainties related to a condition may facilitate the introduction of care planning conversations by encouraging the idea of ‘hoping for the best, but preparing just in case’. Professionals must acknowledge uncertainty in constructive ways that support effective coping, while also ensuring that patients and families receive timely and appropriate supportive and palliative care alongside effective management of this unpredictable and increasingly common life-limiting illness.
